# Terrestrial mammal responses to oil palm dominated landscapes in Colombia

**DOI:** 10.1371/journal.pone.0197539

**Published:** 2018-05-24

**Authors:** Lain E. Pardo, Mason J. Campbell, Will Edwards, Gopalasamy Reuben Clements, William F. Laurance

**Affiliations:** 1 Centre for Tropical Environmental and Sustainability Science (TESS), College of Science and Engineering, James Cook University, Cairns, Queensland, Australia; 2 Grupo de Conservación y Manejo de Vida Silvestre, Universidad Nacional de Colombia. Bogotá D.C. Colombia; 3 Department of Biological Sciences, Sunway University, Bandar Sunway, Selangor, Malaysia; 4 Rimba, Casa Kiara 2, Kuala Lumpur, Malaysia; Sichuan University, CHINA

## Abstract

The rapid expansion of oil palm cultivation in the Neotropics has generated great debate around possible biodiversity impacts. Colombia, for example, is the largest producer of oil palm in the Americas, but the effects of oil palm cultivation on native fauna are poorly understood. Here, we compared how richness, abundance and composition of terrestrial mammal species differ between oil palm plantations and riparian forest in the Colombian Llanos region. Further, we determined the relationships and influence of landscape and habitat level variables on those metrics. We found that species richness and composition differed significantly between riparian forest and oil palm, with site level richness inside oil palm plantations 47% lower, on average, than in riparian forest. Within plantations, mammalian species richness was strongly negatively correlated with cattle abundance, and positively correlated with the density of undergrowth vegetation. Forest structure characteristics appeared to have weak and similar effects on determining mammal species richness and composition along riparian forest strips. Composition at the landscape level was significantly influenced by cover type, percentage of remaining forest and the distance to the nearest town, whereas within oil palm sites, understory vegetation, cattle relative abundance, and canopy cover had significant effects on community composition. Species specific abundance responses varied between land cover types, with oil palm having positive effects on mesopredators, insectivores and grazers. Our findings suggest that increasing habitat complexity, avoiding cattle and retaining native riparian forest–regardless of its structure–inside oil palm-dominated landscapes would help support higher native mammal richness and abundance at both local and landscape scales.

## Introduction

Habitat loss caused by agricultural expansion is one of the main drivers of global biodiversity loss [1][2]. For example, between 1980 and 2000, 55% of new arable land in the tropics came at the expense of intact forest [3]. Within human dominated landscapes, the intensity of the effects of agriculture on native fauna depends on the type of agriculture and landscape and local level factors (e.g. [4][5]). Agriculture growth has been exacerbated by the rapid increase in food demand and the steady rise of global use of fats, oils, and biofuels [6]. Oil palm (*Elaeis guineensis*) cultivation, for instance, has become a major threat to biodiversity in Southeast Asia, where most global production is currently centered [7][8]. Evidence from this region demonstrates that oil palm plantations have negative effects on the abundance and occurrence of a wide range of taxa, including birds, invertebrates, and mammals [9][10][11][12].

In the Neotropics (Latin America), oil palm production is rapidly expanding [13][14]. This expansion is especially evident in Colombia, where the area under oil palm cultivation has increased to nearly 500,000 ha [15], making the country the largest Neotropical oil palm producer. Current government projections suggest oil palm cultivation in Colombia will increase to approximately one million hectares by the year 2020 [16]. If unplanned, this expansion could result in a substantial conversion of natural habitats (e.g., forests, savannas and wetlands), displacement of native wildlife, and disruption of ecosystem functioning [17][18]. However, the likely effects of oil palm on Colombia’s mammals are uncertain as systematic assessments of mammal response to oil palm conversion in Colombia are scarce [19].

The Llanos Orientales region (eastern plains) of Colombia is renowned for its species and ecosystem diversity, comprising large areas of savannas, grasslands, wetlands and riparian forest (known locally as gallery forest) [20]. However, conversion of savannas to agriculture in the Llanos region has increased exponentially from 1970 to 2011 with annual rates of conversion for pasture of approximately 100,000 ha and for oil palm plantations of 5–10,000 ha, especially in the western side of the Llanos [21]. Moreover, the “Altillanura” or high lands of the Llanos has been identified by the government and international agencies as the “new agricultural frontier of Colombia” [19]. The western Llanos is the epicenter for Colombian oil palm production, with approximately 180,000 ha under production [15], though most recent oil palm expansion has predominantly occurred on cropping and grazing lands [22][23]. There is, however, a paucity of studies in the region [24] that can help to understand the biodiversity associated with the region, and the responses of wildlife to growing agriculture and land use change.

Mammals are a good indicator of ecosystem quality or change, given their diversity and the complexity of ecological niches they occupy [25]. Mammals are also important for their role in ecosystem processes, benefits to humans[26][27][28]and their intrinsic and cultural value [29]. At the same time, mammals are one of the most globally threatened taxonomic groups due to habitat loss and fragmentation arising mostly as a product of agricultural expansion [30]. Colombia, for instance, contains the 5th highest level of mammal diversity globally, with 518 species recorded to date [31]. Of these, 43 species are presently threatened with extinction [32]. Therefore, it is important to identify the diversity patterns and responses of Colombian mammal species to oil palm production to ensure the development of effective management strategies, to identify species of concern, and to evaluate the capacity of the country to retain its mammal diversity in the face of rapid palm oil expansion.

In this study, we use an extended camera trapping survey in the western Llanos Orientales- Colombia’s leading oil palm production region, to compare species richness, abundance and composition of terrestrial mammals between oil palm plantations and riparian forests strips, the two most dominant land cover types in the region. Further, we determine the main landscape and habitat correlates driving mammalian species richness, abundance and composition within and between these land cover types in an attempt to identify management practices that may help minimize the impact of this expanding agricultural practice.

## Material and methods

### Study area

**Fig 1:**
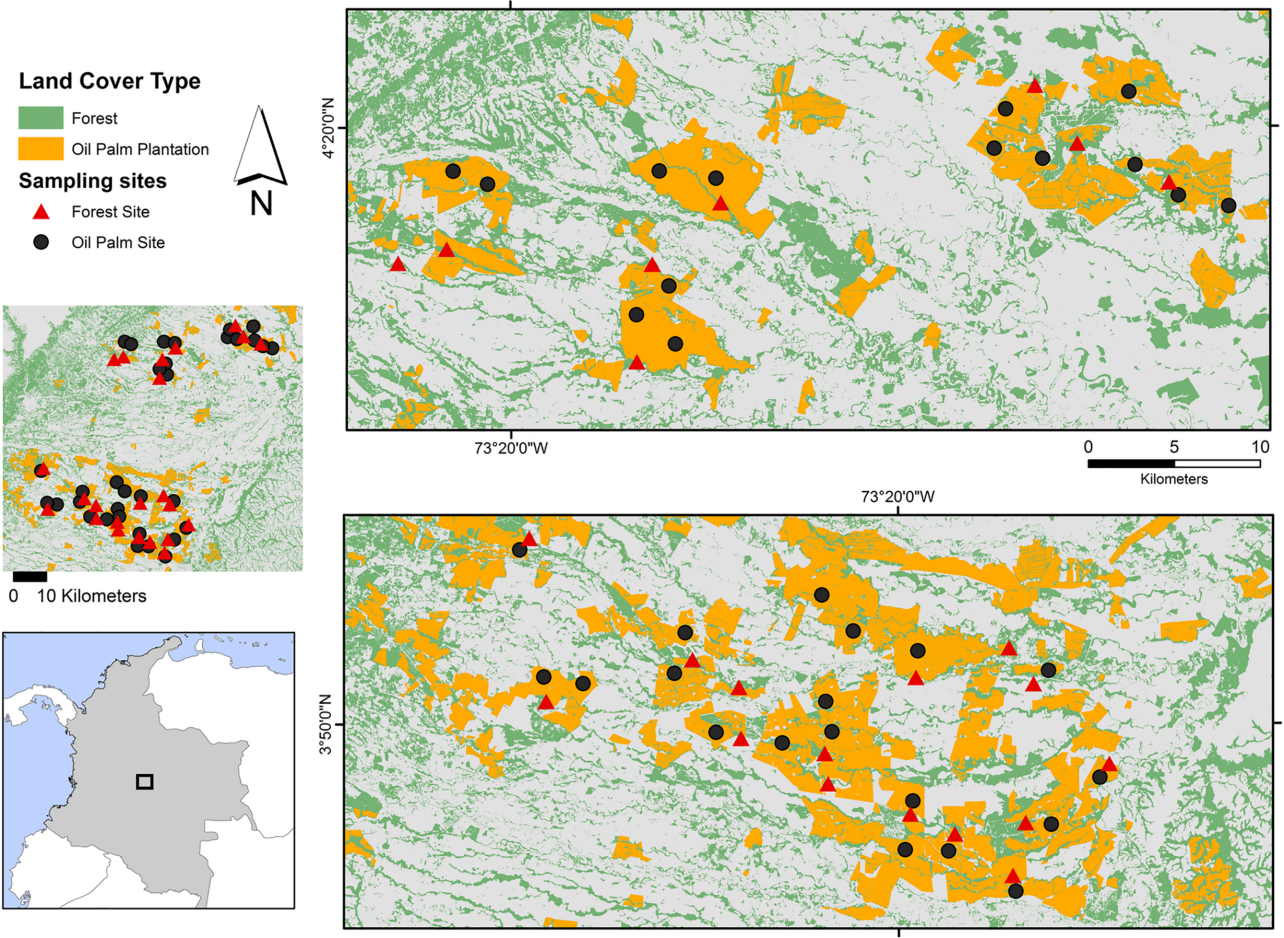
Study area and location of 56 sampling sites in the Department of Meta, Llanos, Colombia.

We conducted this study across a ~2,000 km^2^ area in the rural areas surrounding the towns of Restrepo, Cumaral, Cabuyaro, Acacias, Castilla la Nueva, and San Carlos de Guaroa, in the Department of Meta, situated in the eastern plains or Llanos Orientales region of Colombia, (Fig 1). This area ranges from 194‒394 m.a.s.l. and contains a mosaic of different land cover types including natural ecosystems of differing successional status interspersed by human land uses such as grazing and agriculture. Oil palm production has steadily increased in extent across the area over the last 2 decades and is now the dominant land-use type [18]. Secondary riparian forest strips (or gallery forest) of differing size and age are the predominant remnant native vegetation type and are delineated in this study as young (height < 5 m), intermediate (height ~8 m), and (rarely present) mature secondary forest (height >15 m) [33].

### Sampling design and mammal

We sampled 33 sites in oil palm plantations (hereafter referred to as plantations) and 23 in riparian forests (hereafter referred to as forests) with sampling effort proportionate to the spatial extent of these land covers within the study area (Fig 1). Sampling in plantations was restricted to those planted before 2006 to avoid confounding responses due to plantation age. Sites within each cover type were a minimum of 2 km apart to ensure the spatial independence of samples [34][35] and to encompass the average expected diameter of the common species’ home ranges in the study area [36][37].

We used camera traps to detect medium and large (> 1kg) terrestrial mammals in the dry/transition seasons (~Sept-Mar) across the period September 2014 to January 2016. Since we had a limited number of cameras, the study area was not sampled simultaneously across all sites, and surveys were organized sequentially in different sessions. We used seven cameras to sample each site, as determined by a pilot study that we conducted in the study area (Pardo et al. not publ.). This sampling intensity was implemented to ensure greater sampling completeness compared with traditional practice of using a single camera per site [38]. In riparian forests, camera traps were spaced ~250 m apart along transects to follow the linear nature of the vegetation type, and were set close to animal trails where possible. Due to the homogenous nature of oil palm plantations, cameras in this land cover were spaced similar distances to riparian forests, but were arranged in a zigzag pattern to maximize spatial coverage. We pooled the data derived from the seven cameras at each site into a single sample, which was used as our sample unit for estimating species richness and relative abundance (see below). We identified mammal species using the most recent taxonomic classification of Colombian mammals [31].

Cameras (Reconyx HC500 Hyperfire^TM^, United States [US]) were active for a minimum of 30 days at each site and were configured according to the following criteria: high sensitivity, one-second intervals between consecutive photographs (3 per trigger), no delay or quiet period between triggers, a minimum distance of 1.5 m from an animal’s potential path, and a height of 25‒30 cm depending on the terrain. All cameras were fixed to trees or wooden poles (in the case of cameras inside plantations) with a steel security cable (Python^TM^, US). Arboreal and other species not likely detected by camera trap were recorded opportunistically by direct observations, but were not use for analysis.

This research was conducted in compliance with the Australian Code of Practice for the Care and Use of Animals for Scientific Purposes, 7th Edition, 2004 and the Qld Animal Care and Protection Act, 2001. This study received animal ethics approval from Animal Ethics Committee of James Cook University. The owners of the land and the oil palm companies at each site gave permission to conduct the study on their lands. No specific permissions were required for these locations/activities. The field studies did not involve handling/manipulation of endangered or protected species.

### Species richness and relative abundance

To estimate species richness, we first computed species accumulation curves using EstimateS [39]. To eliminate the influence of the order in which each sample (days) was added, we randomized sample order (n = 1,000). The number of mammalian species was estimated using the Chao 2 (S_est_) estimator of richness [40] a non-parametric estimator that uses incidence data to avoid problems related to detection probabilities and abundance estimation. For each site, we calculated the estimated sampling completeness (ESC) by dividing the number of observed species (S_obs_) over the estimated number of species (S_est_), and then expressed the result as a percentage. A sample-based rarefaction curve was then used to evaluate the effectiveness of the sample effort in R package *vegan* [41][42], and to evaluate if the difference in species richness was significantly different between both land covers (i.e. by examining the confidence intervals of the curve). We tested for potential spatial autocorrelation in the predictors to prevent an inflation of type 2 errors using Moran’s I coefficient within the SAM software V4.0 [43]. In all instances (classes of the correlogram), Moran’s I was not significant (p >0.05).

We used capture frequencies of individual species as a proxy for a relative abundance index (e.g. [44]). This index was calculated as the number of independent photographs divided by the sampling effort x 100. Only detections of the same species taken at periods of greater than 30 minutes were considered as independent [44]. Sample effort was defined as the sum of the number of days that the cameras were active within each transect (camera days). The relative abundance index offers a good alternative when the identification of individuals of species is impossible, and serves as a way to evaluate the structure of the assemblage in terms of commonness and rarity [45], or as a surrogate for “intensity of use”, but should not be used as a measure of abundance or density *sensu stricto* (see [46]).

### Landscape and habitat covariates

#### Landscape covariates

We selected five landscape covariates previously shown to influence mammal richness and composition: 1) percentage of forest [47]; 2) distance to roads (m); 3) distance to towns (m) (e.g. [48]); 4) land-cover/land-use types (hereafter refered to as land-cover type): plantation versus forest [8]; and 5) the Normalized Difference Vegetation Index (NDVI) [49].

To quantify the percentage of forest at each site, we created a 500 m-radius buffer around each camera within each individual transect and then merged the buffers into one single area for analysis. Distance to road and towns was calculated as the average Euclidean distance (m) to the nearest road or town (respectively) for all cameras within the site. These measurements were all obtained using Quantum GIS 2.0.1 [50]. Spatial information for the plantations was supplied by the National Federation of Oil Palm Growers (FEDEPALMA) and Land cover maps acquired from the official ecosystems dataset for Colombia [51]. To identify forested areas, we used data from CLASlite classification [52] which provide improved accuracy and more recent assessment of forest cover. To validate the geographic information available, we also used field notes, Google Earth imagery®, and aerial photographs taken by LEP from a flight over the study area (August 2014; S1 Fig). We calculated the NDVI of multispectral data using Landsat 8 images downloaded on January 2016 (https://landsat.usgs.gov/landsat-data-access). NDVI is widely used as a proxy for vegetation productivity and related parameters, such as net primary production, plant biomass, and vegetation density [53]. Its influence on animal distribution and abundances has previously been confirmed, particularly in areas with different land-uses gradients [49][54]. NDVI values range between zero and one with higher values indicating dense green or unstressed vegetation that is relatively high in quality and productivity [53].

#### Habitat covariates

We used different sets of habitat covariates for assessing patterns within plantations and riparian forests. Within plantations habitat variables were related to crop management practices. These included: 1) the presence of undergrowth vegetation (a factor with 2 levels–see S1 Fig): clean (no)-to-low versus medium-to-high understory vegetation; 2) canopy cover (%); 3) distance (m) to the nearest forest patch; 4) palm height (m); and 5) relative abundance of cattle. To calculate relative abundance of cattle, we used the capture frequency (or catch per unit of effort), as we did for wild mammals described in the previous section. In the forests, we considered four habitat covariates commonly used as a proxy of vegetation structure in natural vegetation systems: 1) canopy cover; 2) tree abundance; 3) diameter at breast height (DBH); and 4) tree height; following the methods of Albesiano and Rangel [55]. We measured these covariates using a 10 x 10 m quadrat around each camera location within the transect, and then obtained a single value for the transect by averaging these measurements for each site.

### Statistical analyses

#### Influence of landscape and habitat variables on species richness

We evaluated the influence of landscape and habitat covariates on mean mammalian species richness using individual Poisson generalized linear mixed models (GLMMs) with habitat and landscape variables as fixed factors, and site as random factor. Prior to model generation, we checked for correlated predictor variables following the protocol of Zuur [56]. To prevent undue influence of measurement unit on any explanatory variables, all explanatory variables were standardized (x‒mean(x))/SD(x)). Standardizing in this manner has the additional benefit that the effect sizes of all variables can be directly compared via model coefficients [56]. We used observed, rather than estimated species richness, as estimated richness had very high confidence intervals in some oil palm sites. Nevertheless, observed and estimated values were very similar and the correlation between the estimated richness and observed richness was high (Spearman’s *r* = 0.91). Primates were not used in any of the analysis because camera traps are not a suitable technique for arboreal animals.

We generated models with all valid combinations of the covariates without interaction effects (32 models for landscape covariates, and 16 models for habitat covariates in the 2 land-cover types) and used an information-theoretic approach to determine the most parsimonious model based on Akaike’s information criterion, corrected for small sample size (AICc) [57]. We used a model-averaging approach when more than one plausible model (i.e., Δ AICc <7) was identified, or when the evidence ratio in support of the “best” model was low. The relative importance of each variable was then assessed by summing the Akaike Weight (*ω*_*i*_) of all plausible selected models containing that variable [57]. Analyses were conducted using the R packages *lme4* [58] and *MuMIn* [59].

#### Influence of landscape and habitat variables on abundance and composition

Analyses of abundance and composition were undertaken in two separate procedures. First, we used a Non-metric Multidimensional Scaling (NMDS) ordination based on Bray Curtis dissimilarity matrix among sites to visualize overall differences in the structure and composition of the assemblage between oil palm and forest (i.e. the distribution of capture records across species and sites). The NMDS is a flexible technique that uses rank orders to evaluate dissimilarities between different communities instead of absolute distances [60]. This ordination was plotted a using R package *vegan*.

Second, to test for the effect of landscape and habitat covariates on overall community composition and on individual species relative abundances, we used a multivariate version of generalized linear modeling (GLM) via *mvabund* R package [61]. This package allows for quantification of factors affecting composition of the whole assemblage (multivariate) and individual species responses (univariate). For this analysis, we rounded the capture frequencies and used it as the response variable, implementing a negative binomial distribution to account for mean/variance relationships. We used the traitglm function and GLM1path with L1 (LASSO) penalty to predict species abundance as a function of landscape and habitat covariates. This function automatically performs model selection, setting to zero any interaction coefficients that do not help reduce AIC [61][62]. We included all covariates used for the species richness analyses and report the size and direction of the model coefficients as a measure of their importance. Only species recorded from more than 15 observations and at more than three sites were included because preliminary analysis returned very high standard errors of parameter estimates for species below these thresholds.

## Results

### General patterns of species richness and relative abundance

We sampled a total of 12,403 camera days and identified 24 ground dwelling species (23 medium to large sized and one small mouse) and two arboreal monkeys, representing seven taxonomic orders and 16 families (S1 Table). Of the 26 identified species, 24 were in the forest and 19 inside oil palm plantations. In the plantations, species richness per site ranged from 1‒7, while in the forests, it ranged from 9‒14. All species detected inside plantations were also detected in the forest, except for red-brocket deer (*Mazama spp*) and a small mouse, whereas seven species were detected only inside the forest; another 17 species recorded in both riparian forest and oil palm plantations (S1 Table). In addition, three more primate species were recorded opportunistically by direct observations but only in the forests. These were the night monkey (*Aotus brumbacki*), the titi monkey (*Plecturocebus ornatus*), and the howler monkey (*Alouatta seniculus*). The first two are considered vulnerable (VU) by the Colombian national assessment of threatened species [63]

**Fig 2:**
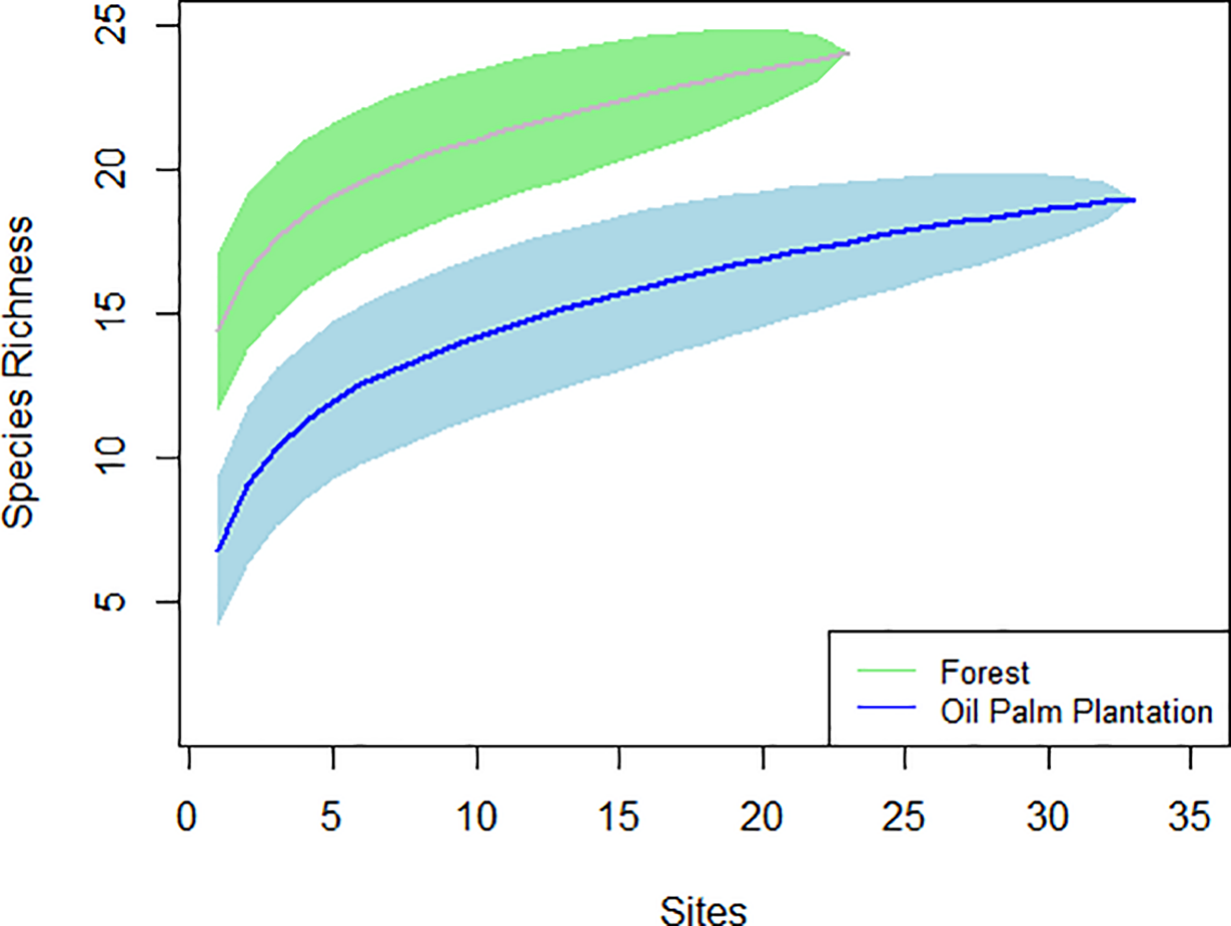
Sample-based rarefaction curves estimating medium and large terrestrial mammal species richness in Llanos, Colombia.

The sampling completeness for mammals in the study area was relatively high, suggesting that the sampling intensity within sites as well as the number of sites captured most of the total species expected in the region (mean = 84%; SD = 15.97). Rarefaction curves showed a representative sample effort with clear asymptotes. Associated confidence intervals of these curves did not overlap, indicating that total richness between plantations and forest was significantly different (Fig 2).

**Fig 3:**
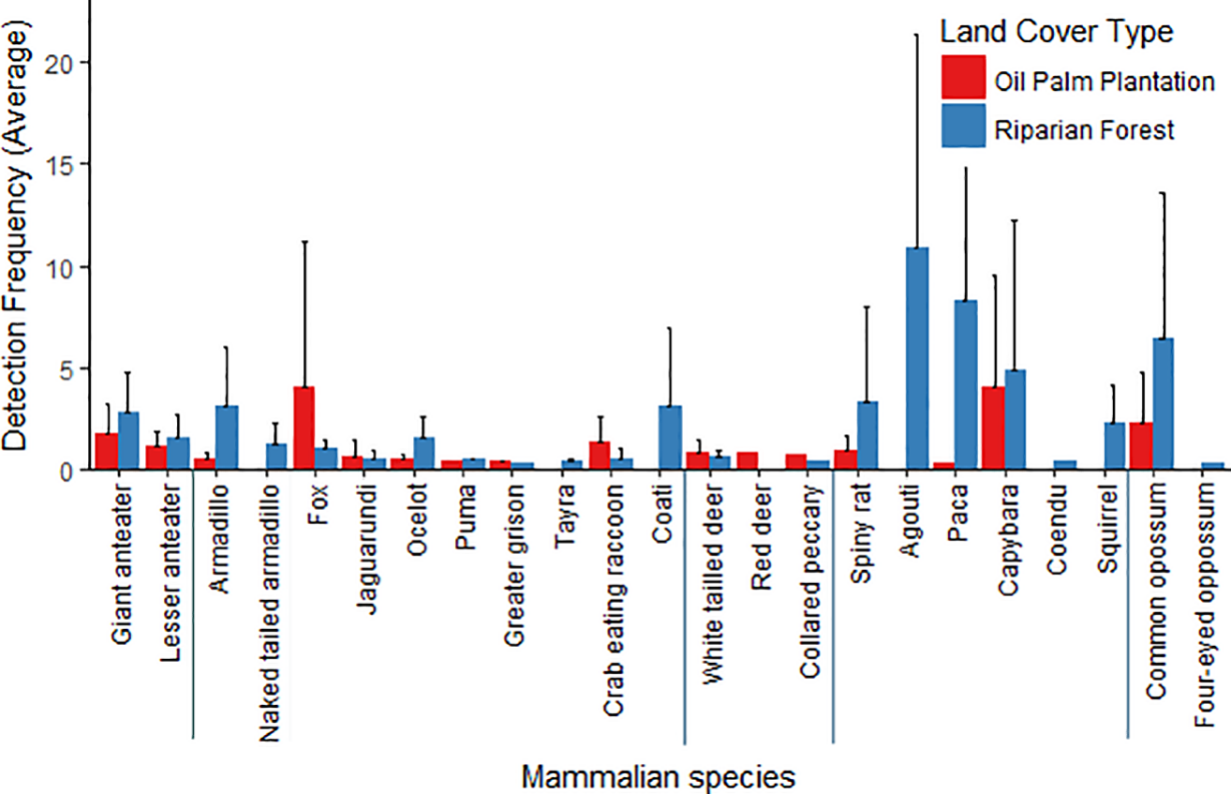
Mean detection/capture frequencies or relative abundances (number of independent photos/sample effort*100) of 23 terrestrial mammal species detected in oil palm plantations and riparian forests in Llanos, Colombia. Bars indicate the upper standard deviation range. Blue lines separate taxonomic orders (from left to right): Pilosa, Cingulata, Carnivora, Artiodactyla, Rodentia, Marsupialia. Note: A mouse species and two species of primates were also detected, but they are not included in this figure because the first could not be identified by camera trap and the latter are not ground dwelling mammals.

The detection frequency (i.e. relative abundance) of the majority of species was low across the study area (Fig 3). Indeed, eight species had fewer than three independent photographs in the entire survey (puma, grison, red-brocked deer, collared peccary, mouse, coendu, tayra, and four-eye opossum; for scientific names refer to S1 Table). Of these the last three were found exclusively inside riparian forests. Plantations had fewer total species detections than forests (582 and 2,085, respectively) (S1 Table). For most other species, relative abundance varied greatly between the two land-cover types and between sites (Fig 3). One species, fox showed clearly higher abundances inside plantations than in riparian forest. Other species also showed higher abundances within plantations, but the magnitude of the difference between habitat types were smaller (i.e. jaguarondi, raccoon and white-tailed deer; S1 Table, Fig 3). All remaining species were detected more frequently in riparian forest sites than in palm plantations (S1 Table, Fig 3). The giant anteater, however, was the only species widely distributed and with relatively high total detections across sites in both plantations and forests (S1 Table).

### Drivers of mammal species richness

#### Landscape level effects

**Fig 4:**
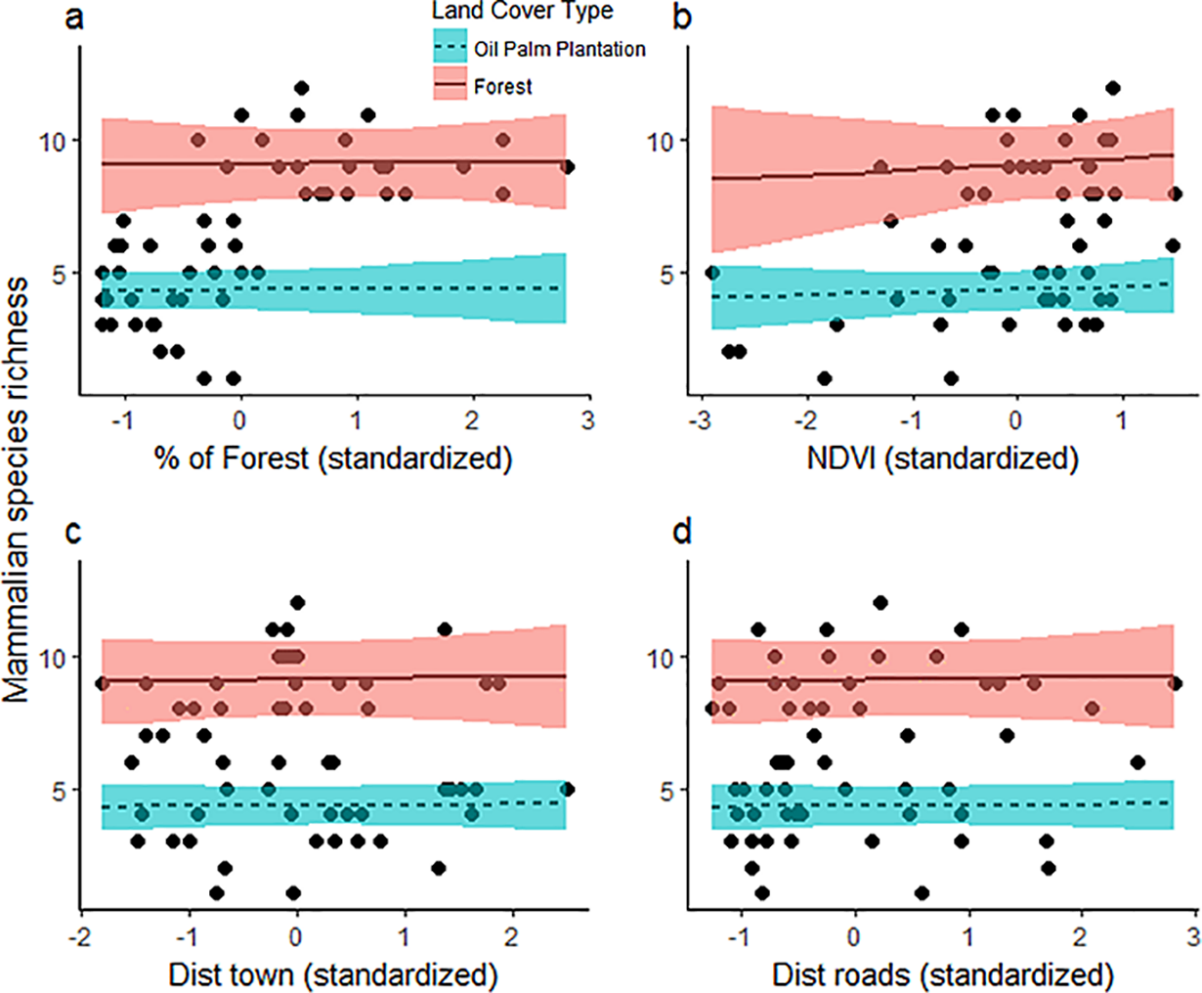
Relationship between mammalian species richness as a function of landscape covariates in Llanos, Colombia: a) percentage of forest, b) NDVI, c) distance to towns, and d) distance to roads, according to land-cover type (oil palm plantations vs forest). The trend lines are predicted values of the GLMM model averaged (holding other covariates constant) and shaded areas represent the 95% confidence intervals. Dotted points represent the actual values of the covariate. Effect of land-cover type is strong, while the slope of continues variables does not show an important effect on species richness.

No single model offered the best explanation for species richness at the landscape level. Results from the averaged model (using 14 suitable candidate models of Δ AICc <7) revealed that land-cover type was clearly the main driver of differences in species richness (∑*ω*_*i*_ = 100%; Table 1). Model averaged coefficients showed that plantations had a negative influence on mammal species richness with site level species richness in plantations 47% lower, on average [(β plantations = -0.74 (SE 0.13)] than that in forests, which showed a high positive influence in determining species richness [β forest = 2.21 (SE 0.08); Table 1)]. All remaining continuous variables exerted weak influence on mammal species richness as indicated by the importance value (combined weight, ∑*ω*_*i*_< 36%) of the averaged variable and effect sizes (Table 1). Percentage of forest, distance to towns, and NDVI all displayed slightly positive effects on species richness, while distance to roads had a slightly negative effect (Fig 4, Table 1; see S2 Table for model ranking).

**Table 1:** Relationship between landscape and habitat covariates and terrestrial mammalian richness in Llanos, Colombia as determined using a GLMM. Estimates correspond to the conditional averaged parameter coefficient and relative importance is based on the *w*AIC -Akaike information criterion.

	Estimate	Adjusted SE	*w*AIC (Relative importance)
**Landscape covariates**			
Intercept	2.21	0.08	
Land-cover type (oil palm plantation)	‒0.74	0.13	1.00
NDVI	0.07	0.07	0.36
Distance to nearest town (km)	0.03	0.06	0.24
Forest (%)	0.02	0.08	0.23
Dist. road (km)	‒0.01	0.06	0.23
**Habitat covariates for oil palm**			
Intercept	1.27	0.20	
Cattle detection frequency	‒0.27	0.15	0.69
Understory vegetation (medium-high)	0.41	0.24	0.55
Height (m)	‒0.13	0.10	0.39
Distance to nearest patch (km)	‒0.11	0.10	0.35
Canopy cover (%)	0.08	0.13	0.26
**Habitat covariates for forest**			
Intercept	2.22	0.07	
Number of trees	‒0.05	0.08	0.22
DBH (cm)	0.05	0.08	0.22
Canopy cover (%)	‒0.02	0.08	0.18
Height (m)	‒0.01	0.09	0.18

#### Habitat level effects in plantations

Similar to the landscape covariates, no single model was identified as demonstrably better than any other, and 15 candidate models were retained based on ΔAICc values (S3 Table). Multimodel averaging indicated that the main predictors of mammalian species richness were relative abundance of cattle (∑*ω*_*i*_ = 69%) and presence of understory vegetation (∑*ω*_*i*_ = 55%). These variables exerted negative and positive effects respectively. Importantly, sites with medium-to-high understory vegetation had 66% more species on average than plantations with no understory vegetation. All remaining variables had weaker effects on species richness [Palm tree height (∑*ω*_*i*_ = 39%), distance to forest patches (∑*ω*_*i*_ 35%, and canopy cover (∑*ω*_*i*_ 26%); Table 1]. Both palm height and distance to forest patches were negative, while canopy cover was positive.

#### Habitat level effects in forests

No single model emerged as a possible driver of mammalian richness inside forest (S4 Table). Contrary to plantations, the model averaged coefficients suggested no evidence of any particular covariate exerting a stronger influence on mammal species richness inside the forests, as shown by their similar contribution. As such, number of trees and DBH had combined effect among models of 22% each, while canopy cover and height had 18% (Table 1); only DBH exerted a positive influence on species richness.

### Drivers of species abundance and composition

#### Landscape level effects

**Fig 5:**
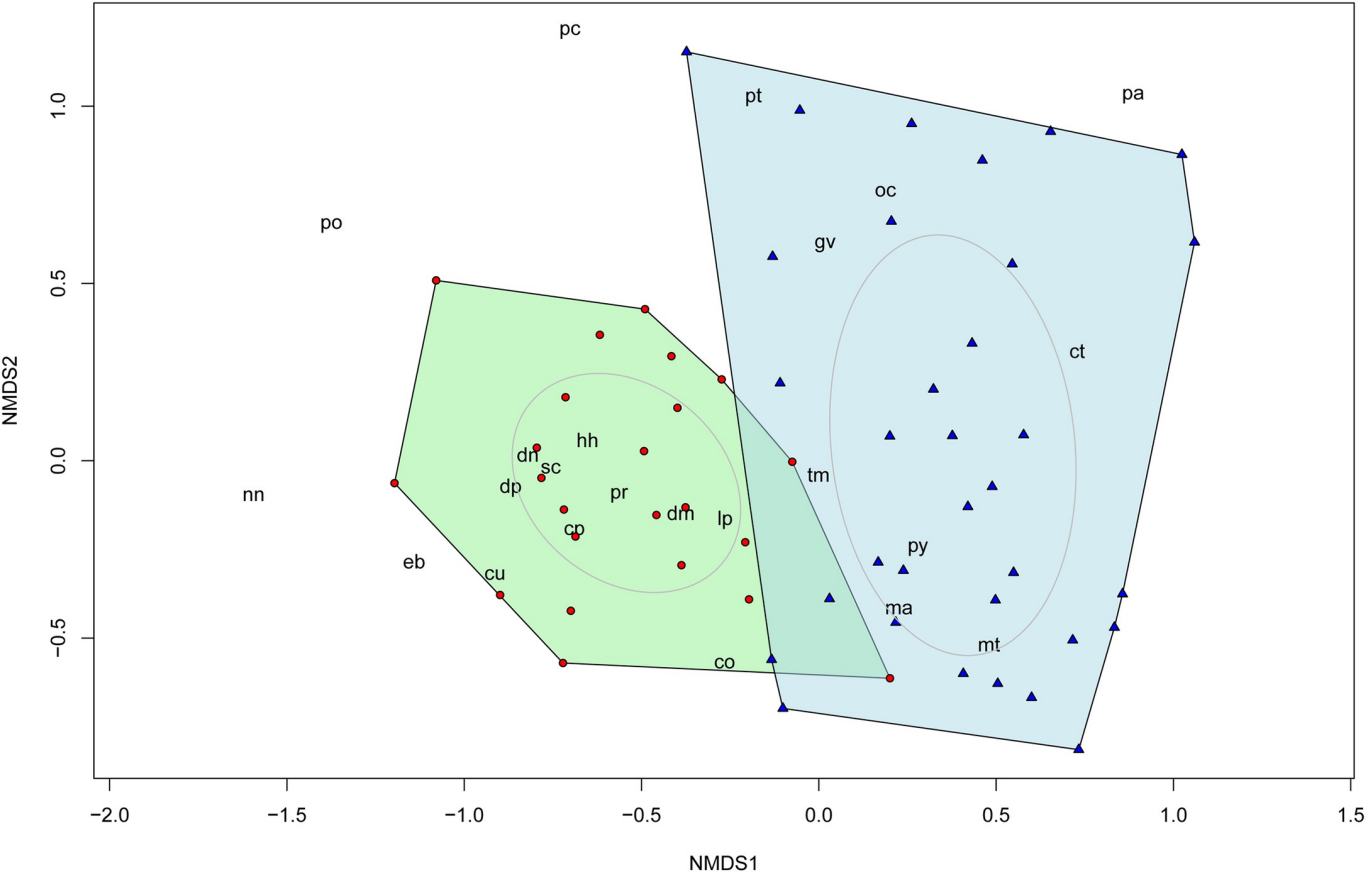
Overall mammal community composition across surveyed sites in oil palm plantations (triangles) and riparian forest (circles). Plot is based on capture frequencies of species using Bray-Curtis non-metric multidimentional analysis (NMDS) (stress = 0.22). Polygons connect the vertices of each cover type and ellipses emphasize the centroids of the community in each land cover. Species outside the boundaries were very rare in the landscape. Codes correspond to the initial letters of the scientific names of each species (refer to S1 Table).

Overall, ordination analysis indicated important dissimilarities in composition between plantations and forest, with plantation sites relatively more scattered and separated from each other (i.e. more different in composition) compared to forest sites (Fig 5*)*. Multivariate GLM confirmed that mammal community composition differed significantly between forests and plantations (Deviance [Dev] = 282.22, Pr (>Dev) = 0.001) Similarly, the percentage of forest and distance to town also had a significant influence on assemblage composition (Deviance [Dev] = 26.04, Pr (>Dev) = < 0.1; Deviance [Dev] = 34.28, Pr (>Dev) < 0.05, respectively; S5 and S6 Tables).

**Fig 6:**
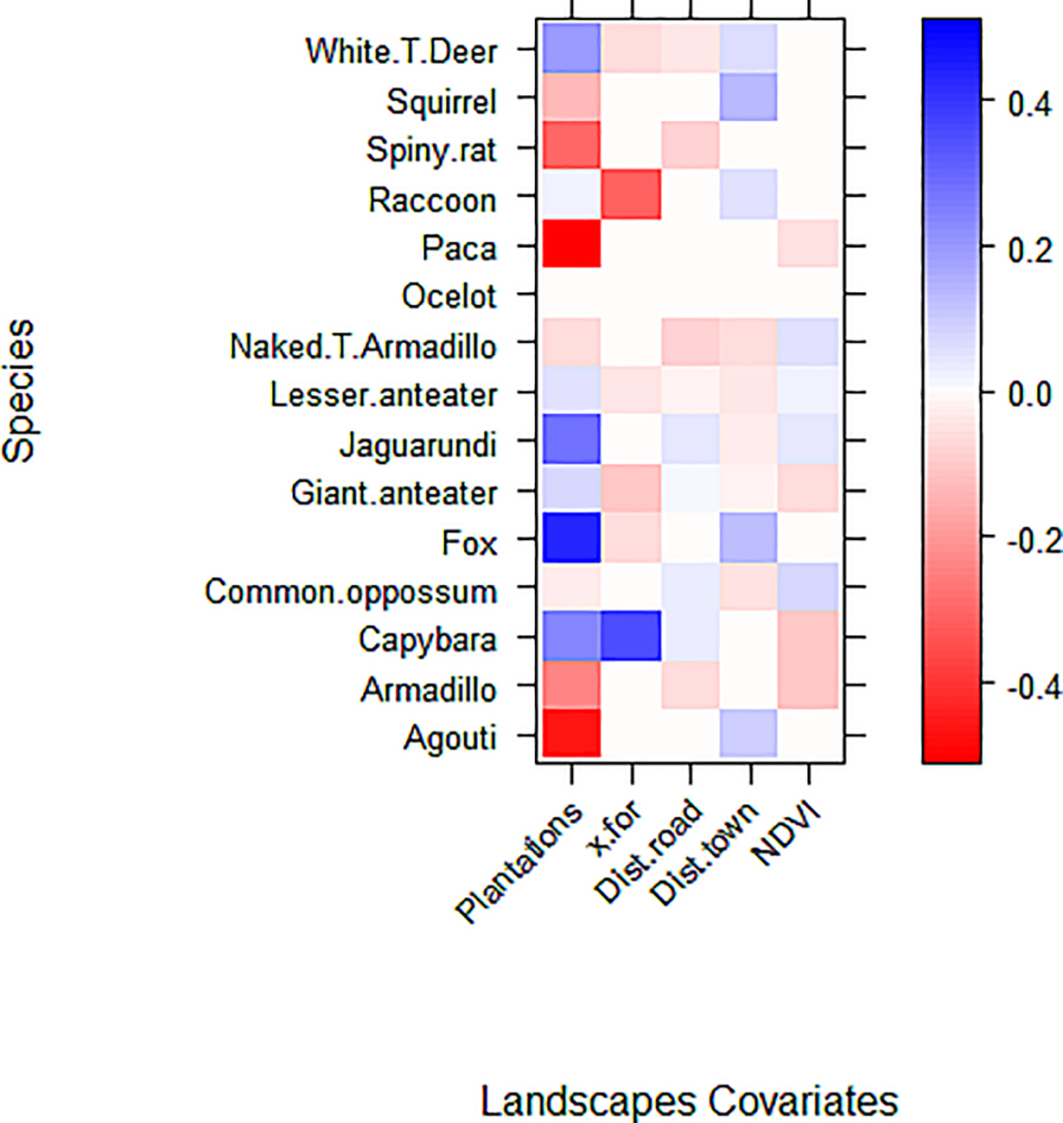
Mammalian relative abundance responses to selected landscape covariates in Llanos, Colombia. Values indicate GLM model coefficients and colors represent the effect sizes on the relative abundance of each species (univariate analysis). Abbreviations: Plantations = oil palm plantation, one of the 2 levels of the categorical variable “cover type” (i.e., riparian forest, and oil palm plantations); x.for = percentage of forests in the 500 m-radius buffer; Dist.road and Dist.town = the average nearest distance to roads and towns (respectively); NDVI: Normalized Difference Vegetation Index. Variables were standardized for direct comparison.

Individual species´ abundance response varied between species. For example, the strongest negative effect of plantations on species abundances were shown by agouti and paca (Fig 6), while the strongest positive effect was found in foxes and jaguarundis (followed by white-tailed deer). Five species (giant and lesser anteaters, ocelots, raccoons, and common opossums) appeared to have neutral responses to land-cover type showing a minimal (positive or negative) influence (Fig 5). Effect of the remaining variables were weak, except for the influence of percentage of forest in the landscape, which had negative and positive effects on capybara and raccoon, respectively (Fig 6).

#### Habitat level effects inside plantations

Understory vegetation, relative abundance of cattle and canopy cover had a significant effect on the community composition (i.e. the overall combined effects of each factor, simultaneously assessed across all species) of mammals within plantations (Deviance [Dev] = 20.88, Pr (>Dev) = 0.045; Deviance [Dev] = 29.57, Pr (>Dev) = 0.002; Deviance [Dev] = 19.87, Pr (>Dev) = 0.043, respectively). Most of the individual species’ coefficients were close to zero, with high standard errors, likely due to the low detections. Therefore, inferences for individual responses inside oil palm should be taken cautiously. However, the detection of rare species, (i.e those with less than three records) such as grison, puma, red-brocket deer, peccary, paca (hence not suitable for statistical analysis), and capybara inside plantations were restricted to sites near to forest (i.e. mean distance among species of 430 m–SD 169 m).

#### Habitat covariates effects inside forests

There was no significant evidence of any habitat variable influencing mammalian community composition inside the forests (DBH = [Dev] = 12.33, Pr (>Dev) = 0.59; tree abundance = [Dev] = 0, Pr (>Dev) = 0.95; canopy cover = [Dev] = 10.44, Pr (>Dev) = 0.75 and tree height = [Dev] = 22.43, Pr (>Dev) = 0.18).

## Discussion

This study aimed to understand the structure and responses of mammal assemblages to an oil palm dominated landscape in Colombia. Our results indicate that richness and composition are significantly reduced in oil palm plantations compared to adjacent riparian forest. However, responses of individual species varied, with the relative abundances of most species responding negatively to oil palm, while other species appeared unaffected or even displayed positive responses to oil palm occurrence, such as mesopredators, insectivores and a grazer. This reduction in diversity is similar to results previously reported for parts of Southeast Asia [9][12][64] and the Brazilian Amazon [65]. However, in contrast to Southeast Asia, reductions in this study occurred to a lesser degree. For example, at individual sites we detected an average of 47% fewer mammal species in plantations than in forests, whereas in some areas of Southeast Asia, fewer than 10% of total local native terrestrial mammals were found to occur in oil palm plantations [9].

The relative little differences in total richness (at the landscape level in particular) between plantations and forest, may be consequence of a long history of landscape transformation, specifically for pasture creation [21][33]. This can further be evident by the reduced abundance of species found. Historical land conversion practices and hunting may have already degraded the forest-dwelling mammals in the study area (i.e. more sensitive species such as giant armadillo–*Priodontes maximus*, danta–*Tapirus terrestris*, or jaguar–*Panthera onca*), decreasing the discernable difference in mammalian species richness between forests and plantations (see also [66][67]). For instance, in the forests we detected only ~43% of the total medium- and large-sized mammal species that may exist in those areas [36]. Further, contrary to oil palm development in South East Asia, where 55% of expansion has occurred at the expense of forests [10], most recent expansion in Colombia has taken place in areas already modified for human land use [23][68]. Therefore, we cannot state that the decrease in present day species can be attributed solely to oil palm expansion in the study area. The lack of baseline data prior the implementation of oil palm limits further evaluations of population trends. Therefore, a monitoring program is recommended.

Our study highlights the importance of secondary forest presence in human-dominated landscapes for biodiversity conservation. In this study, not only were forests important for mammal species richness, but also for their relative abundance. The lack of evidence found for any particular driver explaining richness within riparian forest (see also [69]), suggests that regardless of the forests’ structure, they are fundamental for maintaining mammal species richness in oil palm landscapes; which supports previous findings in secondary forests [70][71]. Nevertheless, the potential of secondary tropical forests to conserve old-growth species is well known to increase over time [72]. Similarly diversity is known to improve in less disturbed and wide riparian forest [73][74]. Therefore, facilitating the natural succession of vegetation from secondary to mature forest in oil palm-dominated landscapes would be an important conservation strategy for native mammal species. Despite not being shown to exert a strong influence as measured against standard statistical “significance’, the fact that richness was positively correlated with NDVI could in part support this suggestion.

Contrary to our predictions, the percentage of forest cover in landscape failed to strongly explain species richness at the landscape level. Although as with NDVI, we found evidence of a positive relationship. This finding corresponds to previous studies (e.g. [65][67]). One possible explanation for this might be that the current mammal assemblage in this area is relatively resilient as long as a minimum forest cover persist. Perhaps as a consequence of the long history of agriculture in the region (see above). However, percentage of forest was important for composition suggesting that changes in mammal populations are more sensitive to percentage of forest than species richness. Similarly, distance to towns was not a relevant factor explaining richness, but appeared as significant for composition. This pattern may be related to the fact that hunting pressures tend to be higher in close proximities to settlements in tropical regions [75].

### Factors improving diversity inside plantations

We identified two factors explaining richness and composition inside oil palm that can be useful for improving management practices to help to sustain mammal diversity (in terms of both richness and composition): reducing cattle grazing pressure, and maintaining a medium-to-high density of undergrowth vegetation. These factors are clearly linked, as cattle reduces undergrowth in plantations through grazing and soil compaction (LEP pers.obs). Grazing has also previously been found to negatively affect the taxonomic and functional diversity of small mammals in Argentina [76]. Furthermore, it has been demonstrated that habitat heterogeneity and complexity helps sustain biodiversity in oil palm plantations [77][78] and other agroecosystems [79][80][81] by improving the complexity of the trophic webs [82]. In this sense, small patches of undergrowth vegetation throughout plantations can improve heterogeneity and provide habitats for numerous animal groups including arthropods, lizards, birds, and snakes (e.g. LEP pers.obs, [83]). Therefore, cattle grazing inside oil palm plantations (see [84]) would not be a good practice as it decreases mammal species diversity. Furthermore, restricting cattle movement along riparian forest would foster forest regeneration [73].

Another potential management practice highlighted by our results was the level of canopy cover within plantation, which had a significant effect on the composition but not in richness. This result implies that plantations with higher levels of canopy cover may be used as a mechanism to increase species movement into and across oil palm plantations. Further study is required to fully examine the effects of canopy cover. One possibility would be to examine assemblage structure between oil palm varieties and/or hybrids that differ in leaf size.

The clear effect of understory vegetation for improving species richness and the potential of manipulating canopy cover inside plantation to promote abundance of species, would support approaches such as “wildlife friendly” production (e.g. [85]) for the Llanos region. These results, differ from studies in Southeast Asia that found poor correlates between biophysical attributes of oil palm plantations or management alternatives and the diversity of mammal species (e.g. [12]). These alternatives would only be effective if the surrounding forest is maintained, as shown here.

### Individual mammal species responses

We assessed individual mammal species responses to potential land-cover transformation (forests to oil palm plantations) as a proxy to evaluate their tolerance or sensitivity to oil palm. The giant anteater, for example, was widely detected in both land cover types across the landscape, and even showed a slightly positive response in abundance to plantations. This positive response confirms that the species can utilize other land use types, such as forest, plantations or pastures [86][87]. The relative high detection rate within oil palm is most likely due to the high prevalence of ants in plantations, as has previously been observed in the Llanos region [84][88]. In this way, giant anteaters may persist in landscapes dominated by oil palm plantations, even though they are the only species categorized as Vulnerable by the IUCN [89] and despite having a specific diet. Nevertheless, conserving this species will also depend on maintaining areas of natural forest as suggested by the high rate of detections we found in forest, and because of their dependency on this habitat for other activities such as resting [90].

Our results for giant anteater contrast those from Mendes-Oliveira et al. [65] who found no evidence for giant anteaters inside oil palm plantations in the Amazon. One possibility for the difference between Mendes-Oliveira et al. [65] results and those presented here could be related to predation pressure. Quiroga et al. [91], for example, found that jaguar has very high preferences for hunting giant anteater, and its capture frequencies can increase up to 70% at sites without jaguars. To what extent detection frequencies of giant anteaters in the plantations of our study area might be related to absence or presence of jaguars is uncertain. However, we did not find jaguar while Mendes-Oliveira et al. [65] did.

Mesopredators in general showed tolerance to plantations. This result is similar to those of Mendes-Oliveira et al. [65] in an Amazon oil palm landscape; though they detected coati inside plantations. Fox and jaguarondi, in particular, were relatively abundant and positively associated with plantations, confirming the ecological flexibility of these species [37]. Furthermore, the phenomenon of increased mesopredator abundance in agricultural landscapes, including oil palm, has been previously documented for numerous species throughout the tropics (e.g. [37][92][93][94]).

The most likely mechanism driving the aparent high relative abundance of mesopredators could be related to sufficient availability of resources through bottom-up effects [95]. We hypothesize then, that oil palm can promote the presence of potential prey, such as lizards, frogs, small rodents, snakes, birds, and arthropods, which has been reported in oil palm ecosystems (e.g. [83][84][96][97][98]). In this way, these mesopredators could be acting as biological control agents for potential pest species [82] and thus potentially assisting plantation management. However, it is unknown whether an increase in mesopredator abundance (especially foxes) in oil palm plantations may result in other unintended outcomes, such as an increase in the predation of local fauna (see [99][100]). Fox diet, though, can also include a wide variety of seed and fruits, including some Neotropical palm fruits [101]. Therefore, it will be important to understanding the trophic relationships occurring in oil palm landscapes.

Overall, numerous terrestrial mammal species were found in both plantations and riparian forests, probably due to the historical context of land use in the study area (apart from likely hunting pressures), which could have limited present-day community to generalist and more ecologically flexible species [67]. However, the highly uneven distribution of species-specific abundances within each land cover type, and the influence of some variables on composition but not in richness, suggest caution for use of mammal species richness as the sole indicator of mammal response to land-use change. For example, in this study seven (29%) of the species detected inside the plantations were recorded from three or fewer individual photographs. Thus, we recommend a concurrent measurement of other metrics that complement richness (e.g. relative abundance) when assessing the effects of land-use change on mammals, as well as considering historical context of land use transformation. From a landscape perspective, this finding may suggest that the matrix (oil palm) is permeable in different degrees to some species, allowing them to move through oil palm on their way between preferred habitat sites, such as forest. However, it is important to note that rare species detected inside the plantations were restricted to sites near to forest (i.e. maximum ~430 m), which is concordant with previous findings in oil palm landscapes [12][65].

### Conservation implications and future scenarios

Certification schemes for sustainable agriculture (e.g., the Roundtable on Sustainable Palm Oil—RSPO; https://www.rspo.org) have traditionally focused on identifying well-conserved areas (e.g. primary forests), or endangered species within production landscapes. However, within the study area in the Llanos region, none of these features are present, or are only present in places far from the study area. This may limit conservation strategies across the Llanos, as they would be considered of “low conservation value”, likely discouraging initiatives from farmers. Our findings suggest that maintaining secondary riparian forests, regardless of their structure, is a fundamental strategy for the conservation of mammal communities. The long history of land use transformation in the western Llanos has reduced these corridors to critical levels (e.g. [33]). Therefore, for this region (and perhaps regions with similar characteristics) an alternative way to encourage conservation is to focus on restoration of riparian forest strips and introduce elements of landscape design inside plantation, such as the maintenance of undergrowth vegetation along with the reduction of cattle as shown in this study. In this sense, enforcing stricter regulation of the minimum legal widths for vegetation buffer zones (see [102]) will be key for restoring mammal assemblage and facilitate the re-colonization or even re-introduction of forest-specialist species.

On the other hand, we only detected one species of conservation concern, the giant anteater, which was frequently detected in oil palm landscapes. Therefore, a question that remains is, which species should be prioritized? Most species were not categorized as conservation concern, which under present certification schemes may be considered irrelevant for conservation (see [103]). We, therefore, suggest that certification schemes may need to be directed away from the identification of only high conservation remnant vegetation and/or listed endangered species, to include appreciation that all lands can provide some conservation value. As a matter of fact, usually people in the area (e.g. workers, owners) ignore the biodiversity surrounding their lands (Pardo *not publ*). Maximize diversity, and their conservation, at local scales plays an important role in maintaining regional diversity. In this sense, our results highlight the potential contribution of privately owned lands toward conserving regional mammal biodiversity. This is especially important in the context of the Llanos region, considering its lack of legislated protected areas.

If oil palm is mainly replacing pastures and other crops in Colombia [68], a future important study would need to compare the diversity of mammals, and other groups, in different types of agriculture (e.g. pastures, rice, sugar cane) with those of oil palm cultivation to clearly understand the potential benefits and negative effects of each alternative. For example, the conversion of pastures to oil palm seems to have more positive than negative effect as diversity levels in pastures are usually lower [84][104].

In terms of the richness and composition of mammal species, we found that the areas of San Carlos de Guaroa and Cabuyaro warrant special attention. In these zones, we detected rare and ecologically important species, such as the puma (see [105] for details), tayra, coati, and peccary, among others. Furthermore, because of its proximity to relatively undisturbed savannas, San Carlos is an important area for habitat connectivity. Finally, the fact that this zone is relatively close to both “Corridor Meta-Casanare” and “Alto Rio Meta,”–two priority conservation areas suggested for the Orinoquia region [20]–makes it an important area for regional mammal conservation.

Oil palm development provides social benefits in Colombia, and plays an important role as source of employment [106]. Therefore, engaging relevant stakeholders is vital to balancing socioeconomic and environmental goals. This is particularly challenging in the face of the likely future developments in isolated areas and natural savannas in Eastern Llanos [18][19]. Development in these areas is partly driven by government incentives and corporate investments, but is also a response to the cessation of internal armed conflict which has allowed access to previously inaccessible areas [107]. If well managed, oil palm can contribute to the sustainable development of Colombia (see [108]). Recognizing the potential contribution of degraded lands and the implementation of better practices, such as those resulted from this study, would be a good incentive to promote conservation across oil palm landscapes, where highly threatened species or pristine lands are not always present. The future of tropical forest biodiversity in a human-modified world may depend on how well humans know and manage the matrix [109], which, in Colombia’s western Llanos, predominantly comprise oil palm plantations.

## Conclusions

This study provides the first comprehensive analysis of the landscape- and habitat-level effects of oil palm cultivation on terrestrial mammals in Colombia. We found that oil palm plantations supported significantly fewer mammal species and different composition than riparian forests. However, we identified that some species, particularly mesopredators, anteaters, and deer were relatively common in oil palm plantations. We found that secondary riparian forests have a fundamental role in mammal conservation in this landscape, regardless of its structure or area. Therefore, if oil palm expansion occurs at the expense of remnant riparian vegetation there will be drastic deleterious consequence for mammal species in the Llanos region. Based on our results, we recommend that to maintain and increase native mammal diversity inside the plantations, oil palm growers should promote undergrowth vegetation and avoid cattle presence inside plantations, along with respecting designated buffer areas that allow for the conservation and restoration of riparian forests. The present-day assemblage in the study area was limited to relative resilient species. In the absence of pristine or highly threatened species, we suggest the development of new ways of recognition for implementation of good practices that could promote the conservation value and awareness of degraded landscapes.

## Supporting information

S1 Fig Images of the study area in the Llanos region of Colombia (Meta Department). a) Aerial photographs (August 2014) of the landscape highlighting riparian forest and oil palm plantations structure. b) Differences in management schemes of understory vegetation in oil palm plantations in Llanos, Colombia. Photo credit: L.E.Pardo.
(PDF)

S1 Table Terrestrial mammal species detected by camera trapping surveys (Aug. 2014 ‒ Dec. 2015) in oil palm plantations and riparian forests in Llanos, Colombia. (DOCX)

S2 Table Model selection output comparing all possible combinations for the effect of variables on mammalian species richness at the landscape level. (DOCX)

S3 Table Model selection output comparing all possible combinations for the effect of variables on mammalian species richness within oil palm plantation level. (DOCX)

S4 Table Model selection output comparing all possible combinations for the effect of variables on mammalian species richness within riparian forest level. (DOCX)

S5 Table Multispecies generalized linear models examining the relationship between the abundance of medium and large terrestrial mammal species and selected landscape covariates in the Llanos region, Colombia. (DOCX)

S6 Table The relationship between mammal species abundance and selected landscape variables in the Llanos region Colombia. Coefficients are from the saturated model using the multispecies generalized linear modelling prior to shrinkage with Lasso penalty (R package mvabund). SE is the standard error of the coefficient. For scientific names and details of the species, refer to S1 Table.
(DOCX)
