# Supplementary material for: Terrestrial mammal responses to oil palm dominated landscapes in Colombia
Source: PLoS One. 2018 May 24;13(5):e0197539. doi: 10.1371/journal.pone.0197539 (PMC5968401; doi:10.1371/journal.pone.0197539)
Supplement: S1 Fig — a) Aerial photographs (August 2014) of the landscape highlighting riparian forest and oil palm plantations structure. b) Differences in management schemes of understory vegetation in oil palm plantations in Llanos, Colombia. Photo credit: L.E.Pardo. (PDF) [file pone.0197539.s001.pdf]

Terrestrial mammal responses to oil palm dominated landscapes in Colombia

Supporting information

S1 Fig. Images of the study area in the Llanos region of Colombia (Meta department). a) Aerial photographs (August 2014) of the landscape highlighting riparian forest and oil palm plantations structure. b) Differences in management schemes of understory vegetation in oil palm plantations in Llanos, Colombia. Photo credit:L.E.Pardo.

a)

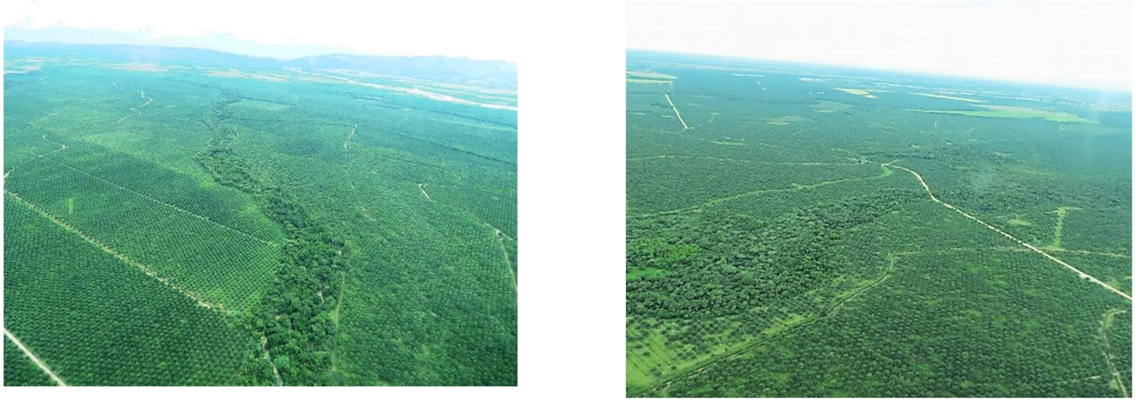

b)

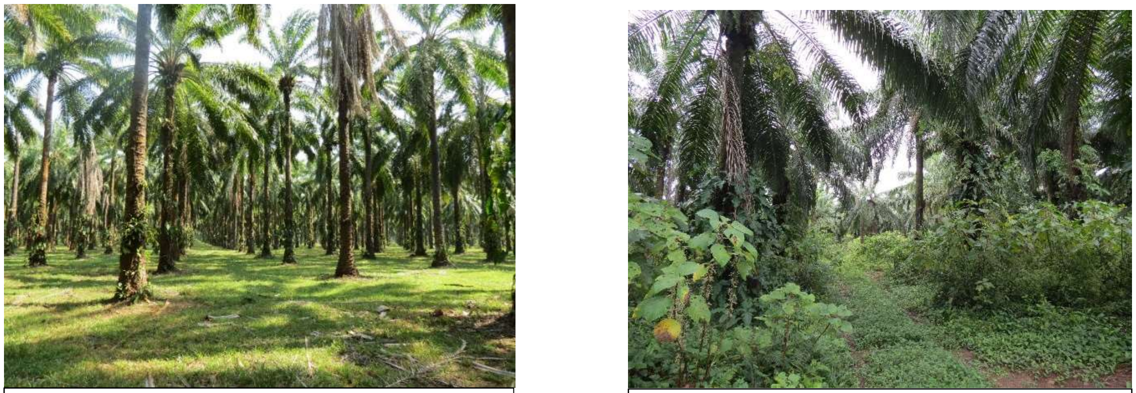

Clean to low understory vegetation	Medium to high understory vegetation
