# Supplementary material for: Terrestrial mammal responses to oil palm dominated landscapes in Colombia
Source: PLoS One. 2018 May 24;13(5):e0197539. doi: 10.1371/journal.pone.0197539 (PMC5968401; doi:10.1371/journal.pone.0197539)
Supplement: S1 Table — (DOCX) [file pone.0197539.s002.docx]

**S1 Table.** **Terrestrial mammal species detected by camera trapping surveys (Aug. 2014 ‒ Dec. 2015) in oil palm plantations and riparian forests in Llanos, Colombia**

|  |  |  |  | **FORESTS** | | | **PLANTATIONS** | | |
| --- | --- | --- | --- | --- | --- | --- | --- | --- | --- |
| **Order/family** | **Common name** | **Scientific name** | **TC** | **Total ind. photos** | **Proportion in survey** | **Total occupied sites** | **Total ind. photos** | **Proportion in survey** | **Total occupied sites** |
| **Pilosa** |  |  |  |  |  |  |  |  |  |
| Myrmecophagidae | giant anteater | *Myrmecophaga tridactyla  ^mt^* | I | 116 | 5.56 | 19 | 123 | 21.03 | 30 |
| Myrmecophagidae | lesser anteater | *Tamandua tetradactyla ^tt^* | I | 65 | 3.21 | 20 | 39 | 6.67 | 16 |
| **Cingulata** |  |  |  |  |  |  |  |  |  |
| Dasypodidae | armadillo | *Dasypus novemcinctus  ^dn^* | I* | 119 | 5.71 | 16 | 4 | 0.68 | 3 |
| Dasypodidae | naked tailed armadillo | *Cabassous unicinctus ^cu^* | I | 25 | 1.20 | 9 | ND |  |  |
| **Carnivora** |  |  |  |  |  |  |  |  |  |
| Canidae | fox | *Cerdocyon thous ^ct^* | MO | 14 | 0.67 | 6 | 226 | 38.63 | 26 |
| Felidae | jaguarundi | *Puma yagouaroundi ^py^* | MC | 6 | 0.29 | 5 | 20 | 3.42 | 13 |
| Felidae | ocelot | *Leopardus pardalis ^lp^* | MC | 57 | 2.73 | 16 | 15 | 2.56 | 12 |
| Felidae | puma | *Puma concolor ^pc^* | T | 2 | 0.10 | 2 | 1 | 0.17 | 1 |
| Mustelidae | greater grison | *Galictis vittata ^gv^* | MC | 1 | 0.05 | 1 | 2 | 0.34 | 2 |
| Mustelidae | tayra | *Eira Barbara ^eb^* | MO | 2 | 0.10 | 2 | ND |  |  |
| Procyonidae | crab eating raccoon | *Procyon cancrivorus ^pa^* | MO | 6 | 0.29 | 4 | 16 | 2.74 | 7 |
|  |  |  |  |  |  |  |  |  |  |
| Procyonidae | coati | *Nasua nasua ^pn^* | MO | 15 | 0.72 | 2 | ND |  |  |
| **Artiodactyla** |  |  |  |  |  |  |  |  |  |
| Cervidae | white-tailed deer | *Odocoileus cariacou ^oc^* | HG | 8 | 0.38 | 6 | 22 | 3.76 | 12 |
| Cervidae | red brocket deer | *Mazama spp ^ma^* | HG | ND |  |  | 2 | 0.34 | 1 |
| Tayassuidae | collared peccary | *Pecari tajacu ^pt^* | HO | 1 | 0.05 | 1 | 1 | 0.17 | 1 |
| **Rodentia** |  |  |  |  |  |  |  |  |  |
| Echimyidae | spiny rat | *Proechimys spp ^pr^* | HR | 182 | 8.73 | 21 | 3 | 0.52 | 2 |
| NN | mouse | *not identified ^nn^* | HR |  |  |  | 2 | 0.34 | 1 |
| Dasyproctidae | agouti | *Dasyprocta fuliginosa ^df^* | HR | 425 | 20.38 | 18 | ND |  |  |
| Cuniculidae | paca | *Cuniculus paca ^cp^* | HR | 469 | 22.49 | 23 | 1 | 0.17 | 1 |
| Caviidae | capybara | *Hydrochoerus hydrochaeris ^hh^* | HG | 99 | 4.75 | 8 | 29 | 4.96 | 3 |
| Erethizontidae | coendu | *coendu spp ^co^* | HF | 1 | 0.05 | 1 | ND |  |  |
| Sciuridae | squirrel | *sciurus spp ^sc^* | HR | 44 | 2.11 | 9 | ND |  |  |
| **Didelphiomorpha** |  |  |  |  |  |  |  |  |  |
| Didelphidae | common opossum | *Didelphis marsupialis ^dm^* | O | 288 | 13.81 | 22 | 63 | 10.77 | 12 |
| Didelphidae | four-eyed opossum | *Philander opossum ^po^* | O | 1 | 0.05 | 1 | ND |  |  |
| **Primates** |  |  |  |  |  |  |  |  |  |
| Cebidae | tufted capuchin | *Sapajus apella* | O | 86 | 4.12 | 19 | 3 | 0.51 | 2 |
| Cebidae | squirrel monkey | *Saimiri sciureus* | O | 61 | 2.93 | 15 | 11 | 1.89 | 2 |

All species are LC (least concern) according to the International Union for the Conservation of Nature Red List Categories (IUCN), except for the giant anteater, which is VU (vulnerable). TC=Trophic Category: I=insectivorous, MO=mesopredator/omnivore, MC=mesopredator/obligate carnivore, HG=herbivorous/grazer, HR=herbivorous/granivorous, HO=herbivorous/omnivorous, O=omnivorous, T=top predator/obligate carnivorous, HF=herbivorous/folivorous. Total ind. Photos = sum of independent photos; independence between consecutive pictures was defined as 30 minutes. ND=not detected. Superscript corresponds to the code for Fig 5.
