# Supplementary material for: Terrestrial mammal responses to oil palm dominated landscapes in Colombia
Source: PLoS One. 2018 May 24;13(5):e0197539. doi: 10.1371/journal.pone.0197539 (PMC5968401; doi:10.1371/journal.pone.0197539)
Supplement: S2 Table — (DOCX) [file pone.0197539.s003.docx]

# **S2 Table**. Model selection output comparing all possible combinations for the effect of variables on mammalian species richness at the landscape level.

| **Inter** | **cover** | **dis.road** | **dis.town** | **NDVI** | **% for** | **R^2^** | **df** | **logLik** | **AICc** | **Δ AICc** | **AICω** |
| --- | --- | --- | --- | --- | --- | --- | --- | --- | --- | --- | --- |
| 2.22 | -0.76 | NA | NA | NA | NA | 0.60 | 3.00 | -113.62 | 233.71 | 0.00 | 0.28 |
| 2.20 | -0.72 | NA | NA | 0.07 | NA | 0.60 | 4.00 | -113.05 | 234.88 | 1.17 | 0.15 |
| 2.22 | -0.77 | NA | 0.02 | NA | NA | 0.60 | 4.00 | -113.55 | 235.89 | 2.18 | 0.09 |
| 2.20 | -0.73 | NA | NA | NA | 0.02 | 0.60 | 4.00 | -113.59 | 235.97 | 2.26 | 0.09 |
| 2.22 | -0.77 | -0.01 | NA | NA | NA | 0.60 | 4.00 | -113.60 | 235.98 | 2.27 | 0.09 |
| 2.20 | -0.73 | NA | 0.03 | 0.08 | NA | 0.61 | 5.00 | -112.87 | 236.94 | 3.23 | 0.06 |
| 2.18 | -0.69 | NA | NA | 0.07 | 0.02 | 0.60 | 5.00 | -113.01 | 237.22 | 3.51 | 0.05 |
| 2.20 | -0.73 | -0.01 | NA | 0.07 | NA | 0.60 | 5.00 | -113.03 | 237.25 | 3.54 | 0.05 |
| 2.21 | -0.74 | NA | 0.02 | NA | 0.02 | 0.60 | 5.00 | -113.52 | 238.25 | 4.54 | 0.03 |
| 2.22 | -0.77 | -0.01 | 0.02 | NA | NA | 0.60 | 5.00 | -113.54 | 238.28 | 4.57 | 0.03 |
| 2.21 | -0.74 | -0.01 | NA | NA | 0.02 | 0.60 | 5.00 | -113.57 | 238.33 | 4.63 | 0.03 |
| 2.18 | -0.69 | NA | 0.03 | 0.08 | 0.02 | 0.61 | 6.00 | -112.83 | 239.38 | 5.67 | 0.02 |
| 2.20 | -0.73 | -0.01 | 0.03 | 0.08 | NA | 0.61 | 6.00 | -112.86 | 239.44 | 5.73 | 0.02 |
| 2.18 | -0.70 | -0.01 | NA | 0.07 | 0.02 | 0.60 | 6.00 | -112.99 | 239.69 | 5.98 | 0.01 |
| 2.21 | -0.74 | -0.01 | 0.02 | NA | 0.02 | 0.60 | 6.00 | -113.51 | 240.73 | 7.02 | 0.01 |
| 2.18 | -0.70 | -0.01 | 0.03 | 0.08 | 0.02 | 0.61 | 7.00 | -112.82 | 241.98 | 8.27 | 0.00 |
| 1.80 | NA | NA | NA | 0.12 | 0.25 | 0.46 | 4.00 | -121.86 | 252.51 | 18.80 | 0.00 |
| 1.80 | NA | NA | NA | NA | 0.27 | 0.41 | 3.00 | -124.03 | 254.51 | 20.81 | 0.00 |
| 1.80 | NA | NA | 0.03 | 0.13 | 0.25 | 0.46 | 5.00 | -121.74 | 254.69 | 20.98 | 0.00 |
| 1.80 | NA | 0.01 | NA | 0.12 | 0.25 | 0.46 | 5.00 | -121.83 | 254.85 | 21.14 | 0.00 |
| 1.80 | NA | 0.02 | NA | NA | 0.27 | 0.41 | 4.00 | -123.97 | 256.72 | 23.02 | 0.00 |
| 1.80 | NA | NA | 0.00 | NA | 0.27 | 0.41 | 4.00 | -124.02 | 256.83 | 23.13 | 0.00 |
| 1.80 | NA | 0.02 | 0.03 | 0.13 | 0.25 | 0.46 | 6.00 | -121.68 | 257.08 | 23.37 | 0.00 |
| 1.80 | NA | 0.02 | 0.01 | NA | 0.27 | 0.41 | 5.00 | -123.96 | 259.13 | 25.42 | 0.00 |
| 1.83 | NA | NA | NA | 0.18 | NA | 0.15 | 3.00 | -134.27 | 275.00 | 41.30 | 0.00 |
| 1.83 | NA | 0.04 | NA | 0.17 | NA | 0.16 | 4.00 | -133.92 | 276.63 | 42.92 | 0.00 |
| 1.83 | NA | NA | 0.02 | 0.18 | NA | 0.16 | 4.00 | -134.23 | 277.25 | 43.54 | 0.00 |
| 1.83 | NA | 0.05 | 0.02 | 0.18 | NA | 0.17 | 5.00 | -133.83 | 278.87 | 45.16 | 0.00 |
| 1.84 | NA | NA | NA | NA | NA | 0.00 | 2.00 | -138.96 | 282.15 | 48.44 | 0.00 |
| 1.84 | NA | 0.05 | NA | NA | NA | 0.02 | 3.00 | -138.51 | 283.48 | 49.78 | 0.00 |
| 1.84 | NA | NA | -0.01 | NA | NA | 0.00 | 3.00 | -138.93 | 284.33 | 50.62 | 0.00 |
| 1.84 | NA | 0.05 | 0.00 | NA | NA | 0.02 | 4.00 | -138.51 | 285.80 | 52.10 | 0.00 |

Abbreviations: inter= intercept, cover = cover type oil palm and riparian forest (the intercept), Dist.road (km) and Dist.town (km) = the average nearest distance to roads and towns (respectively), % for = percentage of forest in the 500 m radius buffer, NDVI: Normalized Difference Vegetation Index. Variables were standardized for direct comparison. R^2^ = variance explained df =degrees of freedom, , logLik = maximum likelihood function, AICc = Akaike Information Critiria corrected for small samples, Δ AICc: difference in AIC values between each model with the lowest AIC model (best model); AICω: Akaike weight.
