# Supplementary material for: Terrestrial mammal responses to oil palm dominated landscapes in Colombia
Source: PLoS One. 2018 May 24;13(5):e0197539. doi: 10.1371/journal.pone.0197539 (PMC5968401; doi:10.1371/journal.pone.0197539)
Supplement: S3 Table — (DOCX) [file pone.0197539.s004.docx]

# **S3 Table**. **Model selection output comparing all possible combinations for the effect of variables on mammalian species richness within oil palm plantation level.**

| **Inter** | **can.cov** | **dis.patch** | **freq.cattle** | **height** | **und.veg** | **R^2^** | **df** | **logLik** | **AICc** | **Δ AICc** | **AICω** |
| --- | --- | --- | --- | --- | --- | --- | --- | --- | --- | --- | --- |
| 1.17 | NA | NA | -0.23 | NA | 0.37 | 0.31 | 4.00 | -58.89 | 127.21 | 0.00 | 0.11 |
| 1.42 | NA | NA | -0.32 | NA | NA | 0.24 | 3.00 | -60.45 | 127.73 | 0.51 | 0.09 |
| 1.07 | NA | NA | NA | NA | 0.56 | 0.24 | 3.00 | -60.54 | 127.92 | 0.70 | 0.08 |
| 1.41 | NA | NA | -0.29 | -0.13 | NA | 0.29 | 4.00 | -59.32 | 128.07 | 0.86 | 0.07 |
| 1.40 | NA | -0.14 | -0.32 | -0.15 | NA | 0.34 | 5.00 | -58.05 | 128.32 | 1.10 | 0.07 |
| 1.41 | NA | -0.13 | -0.35 | NA | NA | 0.29 | 4.00 | -59.45 | 128.33 | 1.12 | 0.07 |
| 1.21 | NA | NA | -0.22 | -0.10 | 0.31 | 0.33 | 5.00 | -58.30 | 128.81 | 1.60 | 0.05 |
| 1.20 | NA | -0.10 | -0.26 | NA | 0.32 | 0.33 | 5.00 | -58.31 | 128.84 | 1.62 | 0.05 |
| 1.12 | NA | NA | NA | -0.12 | 0.47 | 0.27 | 4.00 | -59.76 | 128.95 | 1.74 | 0.05 |
| 1.13 | 0.11 | NA | NA | NA | 0.45 | 0.26 | 4.00 | -59.97 | 129.38 | 2.16 | 0.04 |
| 1.42 | 0.07 | NA | -0.27 | NA | NA | 0.25 | 4.00 | -60.22 | 129.87 | 2.65 | 0.03 |
| 1.18 | 0.03 | NA | -0.21 | NA | 0.36 | 0.31 | 5.00 | -58.87 | 129.96 | 2.74 | 0.03 |
| 1.07 | NA | -0.06 | NA | NA | 0.55 | 0.25 | 4.00 | -60.33 | 130.08 | 2.87 | 0.03 |
| 1.24 | NA | -0.12 | -0.25 | -0.12 | 0.25 | 0.37 | 6.00 | -57.44 | 130.10 | 2.89 | 0.03 |
| 1.41 | 0.08 | -0.13 | -0.29 | NA | NA | 0.30 | 5.00 | -59.16 | 130.55 | 3.34 | 0.02 |
| 1.41 | 0.02 | NA | -0.28 | -0.13 | NA | 0.29 | 5.00 | -59.31 | 130.84 | 3.63 | 0.02 |
| 1.13 | NA | -0.08 | NA | -0.13 | 0.45 | 0.29 | 5.00 | -59.33 | 130.88 | 3.67 | 0.02 |
| 1.43 | 0.20 | NA | NA | NA | NA | 0.16 | 3.00 | -62.14 | 131.10 | 3.89 | 0.02 |
| 1.15 | 0.07 | NA | NA | -0.09 | 0.41 | 0.28 | 5.00 | -59.51 | 131.23 | 4.02 | 0.02 |
| 1.40 | 0.02 | -0.14 | -0.31 | -0.14 | NA | 0.34 | 6.00 | -58.04 | 131.31 | 4.09 | 0.01 |
| 1.15 | 0.12 | -0.08 | NA | NA | 0.42 | 0.28 | 5.00 | -59.59 | 131.40 | 4.18 | 0.01 |
| 1.21 | 0.04 | -0.10 | -0.24 | NA | 0.30 | 0.34 | 6.00 | -58.25 | 131.74 | 4.53 | 0.01 |
| 1.44 | NA | NA | NA | -0.18 | NA | 0.14 | 3.00 | -62.49 | 131.80 | 4.59 | 0.01 |
| 1.20 | -0.02 | NA | -0.23 | -0.10 | 0.32 | 0.33 | 6.00 | -58.29 | 131.80 | 4.59 | 0.01 |
| 1.43 | 0.15 | NA | NA | -0.13 | NA | 0.20 | 4.00 | -61.29 | 132.00 | 4.79 | 0.01 |
| 1.43 | 0.22 | -0.12 | NA | NA | NA | 0.19 | 4.00 | -61.44 | 132.32 | 5.10 | 0.01 |
| 1.43 | 0.16 | -0.12 | NA | -0.14 | NA | 0.24 | 5.00 | -60.38 | 132.98 | 5.77 | 0.01 |
| 1.44 | NA | -0.10 | NA | -0.20 | NA | 0.18 | 4.00 | -61.82 | 133.07 | 5.85 | 0.01 |
| 1.18 | 0.09 | -0.09 | NA | -0.11 | 0.37 | 0.31 | 6.00 | -58.98 | 133.18 | 5.97 | 0.01 |
| 1.24 | -0.01 | -0.12 | -0.26 | -0.13 | 0.25 | 0.37 | 7.00 | -57.43 | 133.34 | 6.12 | 0.01 |
| 1.45 | NA | NA | NA | NA | NA | 0.02 | 2.00 | -64.66 | 133.71 | 6.50 | 0.00 |
| 1.45 | NA | -0.06 | NA | NA | NA | 0.03 | 3.00 | -64.48 | 135.78 | 8.57 | 0.00 |

Abbreviations: inter= intercept, can.cov =canopy cover, dis.patch =distance to nearest patch of forest, freq.cattle =frequency of cattle, height = height of palm tree, und.veg = understory vegetation with two categories “clear to low” represented by the intercept and “medium high”.Variables were standardized for direct comparison. R^2^ = variance explained df =degrees of freedom, , logLik = maximum likelihood function, AICc = Akaike Information Critiria corrected for small samples, Δ AICc: difference in AIC values between each model with the lowest AIC model (best model); AICω: Akaike weight.
