# Supplementary material for: Terrestrial mammal responses to oil palm dominated landscapes in Colombia
Source: PLoS One. 2018 May 24;13(5):e0197539. doi: 10.1371/journal.pone.0197539 (PMC5968401; doi:10.1371/journal.pone.0197539)
Supplement: S4 Table — (DOCX) [file pone.0197539.s005.docx]

# **S4 Table**. **Model selection output comparing all possible combinations for the effect of variables on mammalian species richness within riparian forest level.**

| **Inter** | **can.cov** | **DBH** | **height** | **trees** | **R^2^** | **df** | **logLik** | **AICc** | **Δ AICc** | **AICω** |
| --- | --- | --- | --- | --- | --- | --- | --- | --- | --- | --- |
| 2.22 | NA | NA | NA | NA | 0.00 | 2.00 | -48.61 | 101.82 | 0.00 | 0.37 |
| 2.22 | NA | 0.05 | NA | NA | 0.02 | 3.00 | -48.34 | 103.95 | 2.13 | 0.13 |
| 2.22 | NA | NA | NA | -0.03 | 0.01 | 3.00 | -48.49 | 104.25 | 2.44 | 0.11 |
| 2.27 | -0.04 | NA | NA | NA | 0.00 | 3.00 | -48.57 | 104.40 | 2.58 | 0.10 |
| 2.22 | NA | NA | 0.01 | NA | 0.00 | 3.00 | -48.61 | 104.47 | 2.66 | 0.10 |
| 2.25 | NA | 0.09 | -0.09 | NA | 0.04 | 4.00 | -48.17 | 106.56 | 4.74 | 0.03 |
| 2.28 | -0.06 | 0.05 | NA | NA | 0.03 | 4.00 | -48.27 | 106.77 | 4.96 | 0.03 |
| 2.22 | NA | 0.05 | NA | 0.00 | 0.02 | 4.00 | -48.34 | 106.91 | 5.09 | 0.03 |
| 2.25 | -0.03 | NA | NA | -0.03 | 0.01 | 4.00 | -48.47 | 107.17 | 5.36 | 0.03 |
| 2.22 | NA | NA | 0.00 | -0.03 | 0.01 | 4.00 | -48.49 | 107.21 | 5.39 | 0.02 |
| 2.27 | -0.05 | NA | 0.02 | NA | 0.00 | 4.00 | -48.55 | 107.32 | 5.51 | 0.02 |
| 2.27 | -0.03 | 0.09 | -0.08 | NA | 0.04 | 5.00 | -48.15 | 109.84 | 8.02 | 0.01 |
| 2.25 | NA | 0.10 | -0.10 | 0.01 | 0.04 | 5.00 | -48.16 | 109.85 | 8.03 | 0.01 |
| 2.28 | -0.06 | 0.06 | NA | 0.01 | 0.03 | 5.00 | -48.27 | 110.07 | 8.25 | 0.01 |
| 2.25 | -0.03 | NA | 0.00 | -0.03 | 0.01 | 5.00 | -48.47 | 110.48 | 8.66 | 0.00 |
| 2.28 | -0.04 | 0.10 | -0.09 | 0.02 | 0.04 | 6.00 | -48.13 | 113.52 | 11.70 | 0.00 |
|  |  |  |  |  |  |  |  |  |  |  |

Abbreviations: inter= intercept, can.cov =canopy cover, DBH =Diameter at Breast Height, height = tree height, trees = tree abundance.Variables were standardized for direct comparison. R2 = variance explained df =degrees of freedom, logLik = maximum likelihood function, AICc = Akaike Information Critiria corrected for small samples, Δ AICc: difference in AIC values between each model with the lowest AIC model (best model); AICω: Akaike weight.
