# Supplementary material for: Terrestrial mammal responses to oil palm dominated landscapes in Colombia
Source: PLoS One. 2018 May 24;13(5):e0197539. doi: 10.1371/journal.pone.0197539 (PMC5968401; doi:10.1371/journal.pone.0197539)
Supplement: S5 Table — (DOCX) [file pone.0197539.s006.docx]

# **S5 Table.** **Multispecies generalized linear models examining the relationship between the abundance of medium and large terrestrial mammal species and selected landscape covariates in the Llanos region, Colombia.**

|  | **Cover** |  | **Forest (%)** |  | **Dist.road** |  | **Dist.town** |  | **NDVI** |  |
| --- | --- | --- | --- | --- | --- | --- | --- | --- | --- | --- |
|  | Dev | Pr(>Dev) | Dev | Pr(>Dev) | Dev | Pr(>Dev) | Dev | Pr(>Dev) | Dev | Pr(>Dev) |
| **Multivariate** | 282.22 | 0.001 | 26.04 | 0.089 | 25.27 | 0.137 | 34.28 | 0.021 | 26.34 | 0.12 |
| **Univariate** |  |  |  |  |  |  |  |  |  |  |
| giant ant eater | 1.924 | 0.535 | 3.279 | 0.669 | 0.95 | 0.968 | 1.208 | 0.919 | 2.028 | 0.883 |
| lesser ant eater | 8.277 | 0.03 | 0.278 | 1 | 1.566 | 0.906 | 3.89 | 0.58 | 5.688 | 0.342 |
| armadillo | 31.647 | 0.001 | 0.119 | 1 | 4.088 | 0.572 | 3.293 | 0.637 | 2.499 | 0.846 |
| naked armadillo | 11.886 | 0.012 | 0.954 | 0.992 | 5.626 | 0.354 | 1.242 | 0.919 | 1.138 | 0.946 |
| fox | 14.752 | 0.002 | 0.071 | 1 | 0.003 | 0.995 | 3.486 | 0.637 | 0.774 | 0.967 |
| jaguarundi | 0.278 | 0.822 | 0 | 1 | 3.326 | 0.644 | 0.997 | 0.919 | 4.668 | 0.517 |
| ocelot | 24.82 | 0.001 | 0.165 | 1 | 0.043 | 0.995 | 1.708 | 0.869 | 0.152 | 0.982 |
| crab eating raccoon | 1.981 | 0.535 | 6.835 | 0.159 | 0.154 | 0.994 | 0.633 | 0.919 | 0.284 | 0.982 |
| white tailed deer | 0.38 | 0.822 | 0.034 | 1 | 2.374 | 0.8 | 0.596 | 0.919 | 2.125 | 0.883 |
| spiny rat | 34.525 | 0.001 | 0.031 | 1 | 3.964 | 0.572 | 0.561 | 0.919 | 1.691 | 0.897 |
| agouti | 46.103 | 0.001 | 0.283 | 1 | 0.049 | 0.995 | 4.284 | 0.554 | 0.195 | 0.982 |
| paca | 69.129 | 0.001 | 0.171 | 1 | 0.309 | 0.994 | 2.188 | 0.825 | 0.289 | 0.982 |
| capybara | 2.757 | 0.483 | 13.518 | 0.005 | 0.398 | 0.994 | 0.159 | 0.919 | 1.835 | 0.897 |
| squirrel | 18.945 | 0.001 | 0 | 1 | 0.252 | 0.994 | 6.018 | 0.316 | 0.398 | 0.979 |
| common oppossum | 14.817 | 0.002 | 0.298 | 1 | 2.171 | 0.813 | 4.015 | 0.576 | 2.574 | 0.846 |

Abbreviations: cover =Land cover type, refers to riparian forest and oil palm plantations, forest (%) = percentage of forest in the 500 m radius buffer, Dist.road and Dist.town = the average nearest distance (m) to roads and towns (respectively), NDVI: Normalized Difference Vegetation Index. Variables were standardized for direct comparison. *Dev = Deviance, analysis conducted in R package mvabund– Wang et al. 2012
