# Supplementary material for: Terrestrial mammal responses to oil palm dominated landscapes in Colombia
Source: PLoS One. 2018 May 24;13(5):e0197539. doi: 10.1371/journal.pone.0197539 (PMC5968401; doi:10.1371/journal.pone.0197539)
Supplement: S6 Table — Coefficients are from the saturated model using the multispecies generalized linear modelling prior to shrinkage with Lasso penalty (R package mvabund). SE is the standard error of the coefficient. For scientific names and details of the species, refer to S1 Table. (DOCX) [file pone.0197539.s007.docx]

# **S6 Table.** **The relationship between mammal species abundance and selected landscape variables in the Llanos region Colombia.** **Coefficients are from the saturated model using the multi-species generalized linear modelling prior to shrinkage with Lasso penalty (R package mvabund*). SE is the standard error of the coefficient. For scientific names and details of the species, refer to S1 Table.**

|  | **Giant.anteater** | | **Lesser.anteater** | | **Armadillo** | | **Naked.armadillo** | |
| --- | --- | --- | --- | --- | --- | --- | --- | --- |
|  | *Coefficient* | *SE* | *Coefficient* | *SE* | *Coefficient* | *SE* | *Coefficient* | *SE* |
| (Intercept) | 1.15 | 0.23 | 0.19 | 0.27 | 0.71 | 0.35 | -1.93 | 0.69 |
| Land Cover Type (oil palm plantation) | -0.97 | 0.37 | -0.95 | 0.43 | -4.89 | 1.24 | -12.69 | 156.02 |
| Forest (%) | -0.36 | 0.19 | -0.13 | 0.21 | 0.18 | 0.27 | 0.37 | 0.40 |
| Dist. Road (km) | 0.08 | 0.12 | -0.23 | 0.16 | -0.60 | 0.27 | -1.26 | 0.65 |
| Dist. Town (km) | -0.16 | 0.12 | -0.25 | 0.17 | 0.25 | 0.26 | -0.32 | 0.55 |
| NDVI | -0.18 | 0.12 | 0.49 | 0.22 | -0.57 | 0.36 | 0.79 | 0.73 |
|  | **Fox** | | **Jaguarundi** | | **Ocelot** | | **Raccoon** | |
|  | *Coefficient* | *SE* | *Coefficient* | *SE* | *Coefficient* | *SE* | *Coefficient* | *SE* |
| (Intercept) | -1.33 | 0.64 | -3.19 | 0.95 | 0.16 | 0.31 | -1.71 | 0.90 |
| Land Cover Type (oil palm plantation) | 2.29 | 0.89 | 0.35 | 1.45 | -3.10 | 0.85 | -1.46 | 1.51 |
| Forest (%) | -0.11 | 0.43 | -0.30 | 0.82 | -0.11 | 0.25 | -2.33 | 1.06 |
| Dist. Road (km) | -0.08 | 0.26 | 0.52 | 0.34 | -0.06 | 0.20 | -0.04 | 0.44 |
| Dist. Town (km) | 0.45 | 0.25 | -0.37 | 0.50 | -0.32 | 0.23 | 0.33 | 0.38 |
| NDVI | 0.24 | 0.24 | 1.19 | 0.72 | -0.12 | 0.30 | 0.28 | 0.48 |
|  | **White.T.Deer** | | **Spiny.rat** | | **Agouti** | | **Paca** | |
|  | *Coefficient* | *SE* | *Coefficient* | *SE* | *Coefficient* | *SE* | *Coefficient* | *SE* |
| (Intercept) | -1.65 | 0.58 | 0.92 | 0.38 | 1.88 | 0.43 | 2.13 | 0.30 |
| Land Cover Type (oil palm plantation) | 0.21 | 0.81 | -3.95 | 0.91 | -16.66 | 169.27 | -16.98 | 174.08 |
| Forest (%) | -0.06 | 0.40 | 0.03 | 0.29 | 0.08 | 0.33 | 0.02 | 0.24 |
| Dist. Road (km) | -0.43 | 0.34 | -0.50 | 0.25 | 0.05 | 0.26 | 0.19 | 0.18 |
| Dist. Town (km) | 0.29 | 0.27 | -0.04 | 0.28 | 0.74 | 0.32 | 0.27 | 0.22 |
| NDVI | 0.46 | 0.34 | 0.51 | 0.41 | 0.22 | 0.48 | -0.23 | 0.34 |
|  | **Capybara** | | **Squirrel** | | **Common.oppossum** | |  |  |
|  | *Coefficient* | *SE* | *Coefficient* | *SE* | *Coefficient* | *SE* |  |  |
| (Intercept) | -2.33 | 0.91 | -0.60 | 0.57 | 1.45 | 0.43 |  |  |
| Land Cover Type (oil palm plantation) | 1.00 | 1.30 | -14.19 | 159.49 | -1.80 | 0.68 |  |  |
| Forest (%) | 2.11 | 0.58 | 0.14 | 0.39 | 0.22 | 0.32 |  |  |
| Dist. Road (km) | 0.25 | 0.38 | -0.01 | 0.28 | 0.14 | 0.22 |  |  |
| Dist. Town (km) | -0.38 | 0.42 | 1.01 | 0.40 | -0.35 | 0.24 |  |  |
| NDVI | -0.60 | 0.42 | 0.41 | 0.63 | 0.46 | 0.28 |  |  |

Abbreviations: Land cover type refers to riparian forest and oil palm plantations, forest (%) = percentage of forest in the 500 m radius buffer, Dist.road (km) and Dist.town (km) = the average nearest distance to roads and towns (respectively), NDVI: Normalized Difference Vegetation Index. Variables were standardized for direct comparison. *Wang et al. 2012
